# Experimental evaluation of genetic variability based on DNA metabarcoding from the aquatic environment: Insights from the Leray *COI* fragment

**DOI:** 10.1002/ece3.11631

**Published:** 2024-07-04

**Authors:** S. V. Turanov, M. A. Koltsova, O. A. Rutenko

**Affiliations:** ^1^ Laboratory of Deep sea Research A.V. Zhirmunsky National Scientific Center of Marine Biology, Far Eastern Branch, Russian Academy of Sciences Vladivostok Russia; ^2^ Chair of Cell Biology and Genetics Far Eastern Federal University Vladivostok Russia; ^3^ Laboratory of Molecular Systenatics A.V. Zhirmunsky National Scientific Center of Marine Biology, Far Eastern Branch, Russian Academy of Sciences Vladivostok Russia; ^4^ Laboratory of Ecology and Evolutionary Biology of Aquatic Organisms Far Eastern Federal University Vladivostok Russia

**Keywords:** aquatic biodiversity, *COI*, eDNA metabarcoding, intraspecific diversity, NUMTs

## Abstract

Intraspecific genetic variation is important for the assessment of organisms' resistance to changing environments and anthropogenic pressures. Aquatic DNA metabarcoding provides a non‐invasive method in biodiversity research, including investigations at the within‐species level. Through the analysis of eDNA samples collected from the Peter the Great Gulf of the Japan Sea, in this study, we aimed to evaluate the identification of Amplicon Sequence Variants (ASVs) in marine eDNA among abundant species of the *Zostera* sp. community: *Hexagrammos octogrammus*, *Pholidapus dybowskii* (Teleostei: Perciformes), and *Pandalus latirostris* (Arthropoda: Decapoda). These species were collected from two distant locations to produce mock communities and gather aquatic eDNA both on the community and individual level. Our approach highlights the efficacy of eDNA metabarcoding in capturing haplotypic diversity and the potential for this methodology to track genetic diversity accurately, contributing to conservation efforts and ecosystem management. Additionally, our results elucidate the impact of nuclear mitochondrial DNA segments (NUMTs) on the reliability of metabarcoding data, indicating the necessity for cautious interpretation of such data in ecological studies. Moreover, we analyzed 83 publicly available *COI* sequence datasets from common groups of multicellular organisms (Mollusca, Echinodermata, Crustacea, Polychaeta, and Actinopterygii). The results reflect the decrease in population diversity that arises from using the metabarcode compared to the *COI* barcode.

## Graphical abstract

Intraspecific genetic variation is crucial for understanding organisms' resilience to environmental changes. This study utilized aquatic DNA metabarcoding to assess genetic diversity within the *Zostera* sp. community in the Japan Sea. By analyzing eDNA samples, we identified Amplicon Sequence Variants (ASVs) in *Hexagrammos octogrammus*, *Pholidapus dybowskii*, and *Pandalus latirostris*. Our findings emphasize the effectiveness of eDNA metabarcoding in capturing haplotypic diversity and highlight the impact of nuclear mitochondrial DNA segments on data reliability.

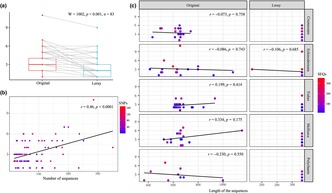

## 1 INTRODUCTION

Studying biodiversity poses several challenges, particularly when assessing intraspecific variability at the DNA level. Data on intraspecific genetic variation of wild populations is important for monitoring and assessing the resistance of organisms to changing environmental conditions and anthropogenic pressures (Hilborn et al., 2003; Schindler et al., 2010). In recent years, data from whole‐genome sequencing have allowed the estimation of population structure, while also revealing features of population demography, gene flow, selection, and introgressive hybridization of specific valuable species, with high accuracy (Leitwein et al., 2020). At the same time, simple measures of genetic diversity for natural populations based on haplotypic variation of individual markers using high‐throughput monitoring would provide exploratory information about the structure of natural populations (see e.g. Adams et al., 2019; Antich et al., 2023; Arribas et al., 2021; Bohmann et al., 2018; Elbrecht et al., 2018; Shum & Palumbi, 2021), which could then be used to guide preliminary recommendations for more elaborate multi‐locus analyses.

Despite the obvious advantages of classic (direct, invasive) monitoring methods for obtaining information on the ecological condition of wildlife, their applicability for some species (e.g., rare and endangered species, or species with low population densities) is limited. They can also produce biased results because of the direct interference of humans, and the methods required for the research process (Field et al., 2019; Minteer et al., 2014; Vucetich & Nelson, 2007). In addition, these approaches are time‐consuming and labor‐intensive (Zemanova, 2020). Therefore, gradual development and transition to alternative, non‐invasive methods is highly needed. Non‐invasive methods for monitoring biodiversity in aquatic environments (Li et al., 2020) include the hydroacoustic technique (Egerton et al., 2018; Lian et al., 2022), image recognition of aquatic organisms using trained neural networks (Goodwin et al., 2021; Siddiqui et al., 2018), and utilizing nucleic acid molecules from the environment (Hering et al., 2018; Jerde et al., 2011; Veilleux et al., 2021). The first two methods help to make real‐time assessments, while DNA from the aquatic environment is being introduced worldwide as an additional tool that can provide insights into the presence of aquatic animals in a particular location, even when their density is low and they are inaccessible by other approaches (Jerde, 2021; Li et al., 2020; Nester et al., 2022; Rees et al., 2014; Veilleux et al., 2021). In contrast to hydroacoustics and neural networks, which are mostly restricted to the species level, the environmental DNA (eDNA) method represents a promising tool for population genetics and phylogeography (Adams et al., 2019; Andres et al., 2021; Elbrecht et al., 2018; Sigsgaard et al., 2020; Tsuji, Maruyama, et al., 2020; Tsuji, Shibata, et al., 2020; Turon et al., 2020).

Studies assessing intraspecific genetic variability via high‐throughput monitoring based on eDNA are largely individualized in a methodological way (Adams et al., 2022; Andres et al., 2021; Elbrecht et al., 2018; Sigsgaard et al., 2017; Tsuji, Maruyama, et al., 2020; Tsuji, Shibata, et al., 2020). Validation and calibration under experimental conditions have only been performed with individual, taxon‐specific markers (Adams et al., 2022; Tsuji, Maruyama, et al., 2020; Tsuji, Shibata, et al., 2020). Without such validation using standardized molecular genetic markers, it is difficult to extend species identification methods to high‐throughput approaches for evaluating the population structure of species in communities. There have been excellent experimental data (Tsuji, Maruyama, et al., 2020; Tsuji, Shibata, et al., 2020) showing the possibility of extracting genetic diversity from hydrobionts via environmental DNA, which are useful for further applications to target species. An additional question arises regarding the possibility of extracting genetic diversity information using standardized markers to assess OTUs (Elbrecht et al., 2018), which has potential to facilitate rapid primary screening of diversity in abundant aquatic species.

Two main approaches are currently used to assess biological diversity based on a bulk reads of high‐throughput sequencing: noise reduction resulting in ASVs or ZOTUs (zero‐radius OTU) and clustering which produce OTUs (operational taxonomic units). OTUs are clusters of similar DNA sequences based on a pre‐defined or flexible threshold of sequence similarity (Edgar, 2018; Kopylova et al., 2016). In order to obtain the identification details, they are assigned to taxonomic groups using reference databases. The OTU can then be used as an equivalent to the morphologically defined species. The main drawback of this approach is that OTUs defined in two different datasets cannot be compared and are dependent on the one in which they are defined (Callahan et al., 2017). ASVs, however, are based on the unique sequences obtained from the sequencing data, without relying on any clustering algorithm and purified from PCR and sequencing artifacts (Callahan et al., 2016). ASVs can also provide more detailed information on the abundance and distribution of rare taxa and can be used to map intraspecific diversity and phylogeographic patterns (Antich et al., 2023; Elbrecht et al., 2018). To maximize the benefits of two approaches these are recommended to be used in combination (Antich et al., 2021).

One of the most commonly used markers in metabarcoding is mitochondrial *COI*, specifically the 313 bp Leray fragment localized within the barcode (Folmer et al., 1994; Geller et al., 2013; Leray et al., 2013). As a first approximation, the use of such a short fragment to assess not only species but also genetic variability of organisms is not reasonable. Indeed, the amount of information on genetic variation increases with the length of the nucleotide sequence, which ultimately increases the accuracy of estimation and prediction. Hence, it would be reasonable to use longer markers for eDNA‐based rapid monitoring, followed by sequencing on third‐generation platforms. However, this particular approach does not work well with DNA from aquatic environments, as it is subject to fairly rapid degradation in daylight (Murakami et al., 2019), up to 36.5% per hour. Although this shows a significant disadvantage of aquatic eDNA, it provides a fundamental opportunity to conduct biodiversity monitoring in dynamics. Another phenomenon that could play a role in combining the drawbacks associated with the use of short molecular genetic markers and the DNA enrichment method via PCR is known as NUMTs (nuclear mitochondrial DNA segments, as described by Lopez et al., 1994). These are fragments of mitochondrial DNA that have been integrated into the nuclear genome. Due to the loss of their original function, such fragments experience alternative evolutionary changes in their new location but can still be amplified using standard primer sets along with (or even instead of) authentic mitochondrial DNA. This, in turn, can lead to inaccurate evaluations of genetic diversity and the real evolutionary paths of species (Cristiano et al., 2012; Hazkani‐Covo et al., 2010; Ožana et al., 2022; Triant & DeWoody, 2007).

NUMTs are also well represented in the metabarcoding *COI* Leray fragment (Schultz & Hebert, 2022), suggesting that NUMTs may compete with mitochondrial DNA for detectability in PCR‐based studies and, theoretically, skew the estimates of authentic ASVs' representation, affecting quantitative measures.

Accordingly, we aimed to consider the possibility of non‐invasive rapid assessment of genetic diversity among abundant aquatic species using DNA from aquatic environments based on metabarcoding of the Leray marker of the *COI* region. To this end, we designed an experiment based on the two artificial communities consisting of abundant, relatively small hydrobiont species that inhabit the *Zostera* sp. seagrass belt communities in the northwestern part of the Japan Sea—Peter the Great Gulf. This is the largest gulf along the Russian coast of the Japan Sea. It is located between two climatic zones, where cold waters of Primorsky and warm waters of North‐Korean currents meet. The bay is characterized by high species diversity and abundance of fish resources (Kalchugin, 2021). The targets for the experiment in this study had to meet a number of criteria: relatively small size, abundant in both collection sites (Vostok and Vityaz bays), similar and easy dietary requirements, able to sustain transportation and long‐term maintenance in common aquaria. In accordance with these criteria, three common species inhabiting the seagrass belts were selected: greenling *Hexagrammos octogrammus* (Pallas, 1814), shrimp *Pandalus latirostris* (Rathbun, 1902), and prickleback *Pholidapus dybowskii* (Steindachner, 1880). This allowed us to simulate natural biodiversity within controlled conditions and implement high‐throughput sequencing together with specific bioinformatic analysis to evaluate the efficacy of eDNA metabarcoding in capturing intraspecific genetic diversity from environmental DNA samples and its susceptibility to the influence of NUMTs.

Moreover, in the present work, we also performed calculations of the genetic variability of the standardized *COI* region. A substantial amount of published sequence data was used to evaluate the applicability of this region for high‐throughput monitoring of the genetic diversity in wild populations of aquatic organisms.

## 2 MATERIALS AND METHODS

### 2.1 Assessment of intraspecific genetic diversity based on aquatic DNA metabarcoding

#### 2.1.1 Collecting hydrobionts and DNA from the aquatic environment

**FIGURE 1:**
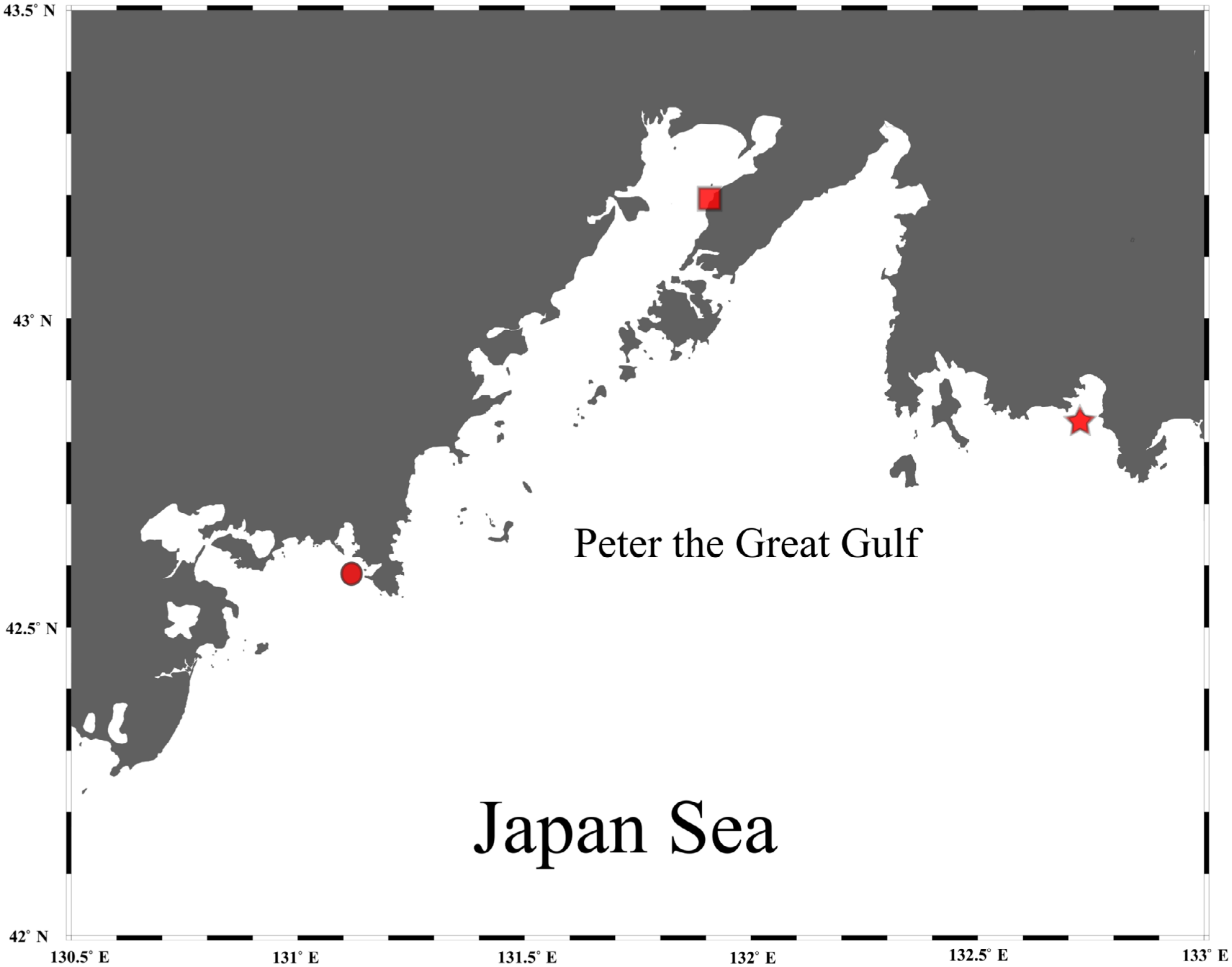
Map of the area where the animals were collected and the experimental work was carried out. Vityaz Bay is marked with a circle, and Vostok Bay is marked with an asterisk. The square marks the location where the animals were placed in the aquarium to form the mock communities (Amur Bay). The map was produced in Ocean Data View (https://odv.awi.de/).

Animals were collected from two locations in Peter the Great Gulf of the Japan Sea (Figure 1): Vostok Bay (three specimens of masked greenling *Hexagrammos octogrammus*, eight specimens of prickleback *Pholidapus dybowskii*, and 16 specimens of shrimp *Pandalus latirostris*), and Vityaz Bay (four, six, and 22 specimens, respectively) in September 2020 using a fish fry net.

#### 2.1.2 Experiment with aquatic animals

The collected hydrobionts were settled in two separate 150 L aquaria of the A.V. Zhirmunsky National Scientific Center of Marine Biology, according to the collection sites of (Figure S1). The aquaria were filled with water from a storage reservoir that is periodically replenished with water from the Amur Bay. Temperature was maintained at 15°C throughout the experiment. The hydrobionts were fed crushed fillets of squid (*Todarodes pacificus* (Steenstrup, 1880)). Shortly, after the introduction to the tanks, one of the greenlings in the Vostok aquarium died and was eaten by the shrimp. Similarly, some of the shrimp were lost in both aquaria. Thus, only a single shrimp remained in the Vityaz aquarium, and eight were in the Vostok aquarium. The number of pricklebacks did not change in either tank. The circulation and filtration of water in the tanks were turned off 1 h before sampling.

Environmental DNA was collected from both aquaria as follows. 900 mL of water was sampled twice using a 150 mL syringe. The entire volume of water was passed through a syringe filter cap (diameter 33 mm, pore size 0.45 μm; PES material) by squeezing water through cap with a modified sealant gun. DNA on the filter was then preserved by passing 1 mL of Longmayer's buffer (Longmire et al., 1997) through the syringe tip, and the inlet and outlet ports were closed with combi‐stopper plugs. The filters were stored at −20°C until DNA isolation.

In addition, we similarly filtered the water from the marine water storage reservoir as a control sample. The aquatic DNA was then collected from the animals individually (Figure S1). Briefly, each hydrobiont was settled into a separate 1.2 L aquarium. Prior to the transfer of the individuals, each aquarium was cleaned with a 10% bleach solution and then rinsed with water from the storage reservoir. To ensure the removal of potential eDNA residues from the mock community after the hydrobiont was introduced, it was rinsed with three volumes (~3.6 L) of water, followed by filling the individual aquarium. After a brief period (30 min to 1 h), the water was sampled and filtered (900 mL per animal), and each animal was placed into a container with a 10% urethane solution for sedation. After sedation, each animal was measured, weighed, and then material was taken for genetic analysis (for fish, a piece of skeletal muscle was cut off from the back; for shrimp, one leg was taken) and preserved in 95% ethanol. In total, 29 individual aquatic eDNA and tissue samples were collected.

#### 2.1.3 DNA isolation and individual genotyping of hydrobionts

Total DNA was extracted from the fixed tissue using a K‐SORB‐100 kit (Syntol, Russia). The isolated DNA was then used to amplify two fragments of mitochondrial *COI* gene—a 313 bp Leray fragment (Geller et al., 2013; Leray et al., 2013; Wangensteen et al., 2018), and a 650 bp fragment (Ward et al., 2005). For the latter, a combination of FishF1 and FishR1 primers was suitable for fish. However, for shrimp genotyping, we used a modified FishF1 primer (5′‐TCAACTAACCACAAAGACATTGGAAC‐3′). The PCR mixture consisted of 5× Taq Red buffer (5 μL), dNTPS (0.5 μL, 10 mM), a pair of primers (0.12 μL, 0.05 mM each), Taq polymerase (0.25 μL, 1.25 units per reaction), 1 μL of DNA solution (20–100 ng), and deionized water to a final reaction volume of 25 μL. The PCR thermal algorithm started with a pre‐denaturation for 10 min at 95°C, followed by 35 cycles consisting of: denaturation at 94°C for 1 min, annealing at 45°C for 1, and 1 min elongation at 72 °C, with a final elongation for 10 min. Amplification success was checked with electrophoresis of the fragments in 1% agarose gel followed by exposure in ethidium bromide solution and visualization under UV. The successfully amplified samples were purified by alcohol precipitation, and the DNA pellet was dissolved in deionized water. Forward and reverse sequencing reactions were performed using purified amplicons together with the corresponding primers used in the PCR according to the manufacturer's instructions for the BrilliantDye™ Terminator Cycle Sequencing Kit (NimaGen, Netherlands). The capillary electrophoresis of the fragments produced by cycle sequencing was performed on an Applied Biosystems Genetic Analyzer 3500. Consensus sequences were assembled from the obtained chromatograms using the Geneious program (Kearse et al., 2012). These sequences were used to search for the homologs in NCBI via the BLAST algorithm (Camacho et al., 2019). The alignment together with the reference sequences selected from the BLAST results and a read frame search using the translation tool was performed in MEGA 7.0 (Kumar et al., 2016). Sequence matrices were then generated for each species separately, and haplotypic variation was assessed in DnaSP v.6 (Rozas et al., 2017). Based on the genotyping results that identified two haplotypes for both *P. dybowskii* and *P. latirostris*, and three haplotypes for *H. octogrammus* (as detailed in Table 1), we selected individual aquatic DNA samples that corresponded to these detected haplotypes of the 313 bp *COI* fragment. For each haplotype, one aquatic DNA sample was chosen for deeper analysis.

**TABLE 1:** Results of genotyping the *COI* mitochondrial fragment of aquatic organisms.

Species	Sampling location	Number of individuals		Number of haplotypes	
		Beginning of the experiment	End of experiment	Leray (313 bp)	Folmer (~650 bp)
*H. octogrammus*	Vostok	3	2	2	2
	Vityaz	4	4	1	3
*P. dybowskii*	Vostok	8	8	2	2
	Vityaz	6	6		
*P. latirostris*	Vostok	16	9	1	1
	Vityaz	22	1	1	1

#### 2.1.4 DNA extraction from syringe filters, COI Leray fragment amplification, sequencing, and read processing

Aquatic DNA was extracted from the syringe filters using an M‐Sorb‐OOM kit (Syntol, Russia) with modification of the manufacture's protocol; at the initial stage, the lysis buffer was heated to 65°C and passed through the filter tip in the opposite direction to the filtration (backflushing method, after [Kesberg & Schleheck, 2013]). The entire volume of the resulting liquid was drained into a clean test tube. Based on the isolated DNA, a 313 bp *COI* fragment was amplified (Geller et al., 2013; Leray et al., 2013; Wangensteen et al., 2018) with three replicates per sample. For each sample, we used a pair of primers with an individual 7‐nucleotide tag at the 5′ end (doubly tagged approach) that were developed in ecotag (Boyer et al., 2016). A negative PCR control was also processed using a separate pair of tagged primers. The reaction mixture included 10 μL of high‐fidelity AmpliTaq Gold 360 Master Mix (Applied Biosystems, USA), 0.5 μL of each (forward and reverse) primer (10 μM), 0.16 μL of bovine serum albumin, 10 ng of DNA and deionized water to a final volume of 20 μL. The PCR thermal cycling profile included preheating at 95°C for 10 min, with 35 subsequent cycles according to the following scheme: 1 min at 94°C, 1 min at 45°C, and 1 min at 72°C. The program concluded with a final elongation at 72°C for 5 min. The results of amplification were checked in the same way as described above. The amplicons were purified using Cleanup S‐Cap (Evrogen, Russia) and normalized (see Elbrecht & Steinke, 2019) before pooling. The volume of PCR negative control reaction was taken as an average of the obtained volume of normalized samples. The normalized amplicons were then combined with the control and sequenced at Novogene (Tianjin, China). The library was created using a PCR‐free NEBNext Ultra II DNA Library Prep Kit for Illumina (New England Biolabs, England) and run on an Illumina NovaSeq 6000 sequencer with 250 bp paired‐end sequencing technology. The reads uploaded to NCBI Sequence Read Archive under SRR22284826 run number.

After removing adapters and pre‐assessing the quality of the reads with fastqc, Spades (Bankevich et al., 2012) was used to correct possible errors. Paired‐end reads were merged into consensus reads using PandaSeq (Masella et al., 2012). The reads were then de‐multiplexed, sorted, and filtered (denoized) using Begum (Zepeda‐Mendoza et al., 2016; Yang et al., 2021; see Supplement A for tags and samples information in Dryad data repository). This step eliminates potential PCR and sequencing artifacts. To extract original haplotypes and all maximally homologous sequences from the obtained ASVs, the following steps were undertaken (see Figure S1). To identify the target sequences, we assembled a local reference library consisting of identified haplotypes (Table 1). The local reference library was then utilized for classifying the ASVs derived from reads based on the micca *classify* command (Albanese et al., 2015), with a similarity threshold of 0.6. The similarity threshold was empirically set to include the maximum number of homologous ASVs to the reference and exclude non‐target DNA (e.g., bacterial DNA). Furthermore, a simple distance‐based neighbor‐joining phylogenetic analysis was conducted using the final set of sequences in MEGA, allowing for the definitive exclusion of non‐target ASVs that did not belong to clusters with target ASVs. To assess the number of reads assigned to each ASV identified in this manner, we employed the micca *otu* command with Swarm algorithm (Mahé et al., 2015). For this, the ASVs identified in the previous step, along with the original haplotypes, were used as a reference for closed_ref clustering of all ASVs with a parameter of ‐d 1.0.

**FIGURE 2:**
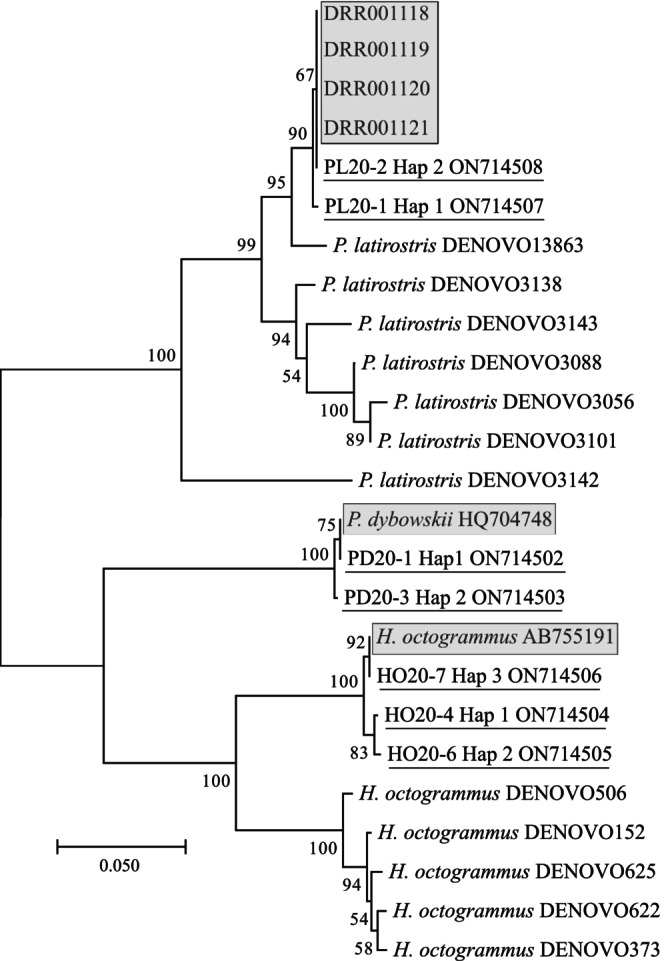
Mid‐rooted phylogenetic NJ‐tree showing the relationships between the original genotyped haplotypes (underlined), reference sequences (highlighted by a frame), and ASVs identified by eDNA sequencing (all others). The sequence matrix is based on the Leray fragment length. The tree was constructed based on uncorrected genetic *p*‐distances. The nodes show the results of the topological robustness analysis, in %.

The information was then summarized in Table 3. The results were further condensed using the LULU package (Frøslev et al., 2017) to test the effect of preserving non‐original ASVs (Table 4). After the ASVs were identified from the local database, it was necessary to clarify their relationships to the original haplotypes. In addition, the *COI* barcode sequences from *H. octogrammus* and *P. dybowskii* species were used to verify the species identity of the haplotypes obtained. Since no reference data are available for *P. latirostris*, we assembled *COI* fragments from reads of four transcriptomes of this species (Kawahara‐Miki et al., 2011) using NOVOPlasty (Dierckxsens et al., 2017). Based on the combined matrix, a phylogenetic neighbor‐joining (NJ) tree was constructed in the MEGA (Figure 2). The robustness of the topology was assessed using 1000 replicates of the non‐parametric bootstrap test. Additionally, the variability of ASVs was evaluated based on uncorrected *p*‐distances (Tables 5 and 6).

**FIGURE 3:**
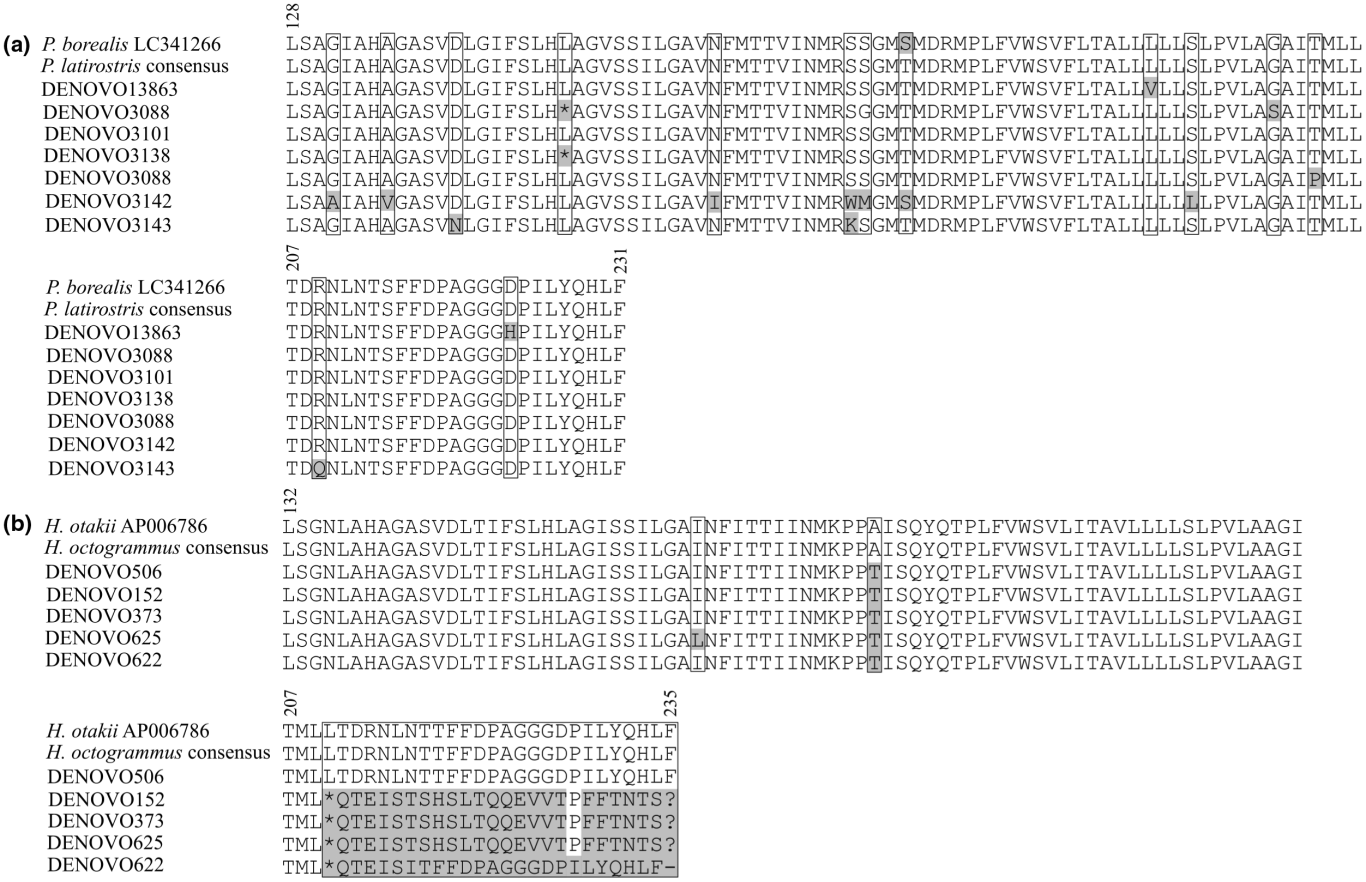
The fragments of the interleaved amino acid sequence alignments of the Leray region produced by translation from nucleotide sequences with corresponding genetic code. The numbers above the matrix indicate the positions of the sites in the complete amino acid sequence of the *COI* gene relative to (a) *P. borealis*, (b) *H. otakii*. Variable sites are indicated by boxes. Shaded areas show amino acids with changes relative to the reference sequence of the species (labeled as “consensus”). The consensus sequences are represented by reference haplotypes and collapsed as they have no variation at protein level. Asterisks indicate stop codon (TAG), dash corresponds to deletion.

To identify potential changes in the reading frame of additional ASVs and their possible classification as pseudogenes (NUMTs), we aligned them along with reference sequences from GenBank using MUSCLE (Edgar, 2004) in MEGA and removed the gaps. The resulting matrices were then translated into amino acid sequences, taking into account the relevant genetic code. The obtained amino acid matrices were visualized (Figure 3) using SeaView (Gouy et al., 2010).

### 2.2 Assessing the genetic variation of *COI* fragments in the population datasets retrieved from GenBank

**FIGURE 4:**
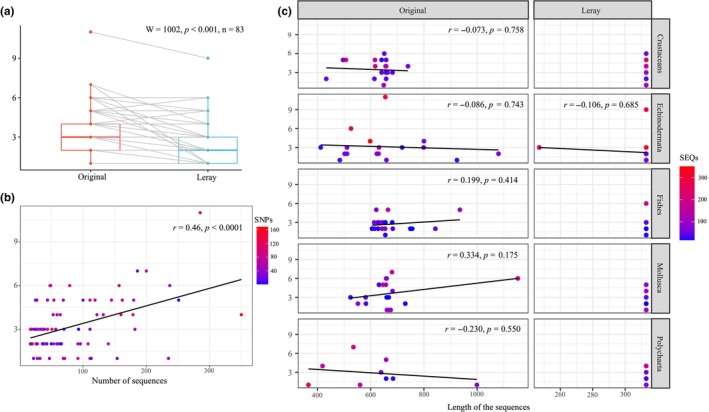
Number of intraspecific clusters estimated for *COI* datasets. (a) Comparison of the average number of clusters between the original (Original) and reduced (Leray) datasets. A Wilcoxon signed rank test was utilized to assess the significance of the decrease in the number of clusters when reducing the size of the fragment (*p* = 5.32e^−9^). (b) The relationship between the number of intraspecific clusters and the number of sequences in the dataset. This analysis is based on the original data. A linear regression line is shown. The color gradient, ranging from blue to red, represents the variation in the number of sites with SNPs. (c) Comparison of the number of intraspecific clusters across taxonomic groups, considering sequence length and the number of sequences in the dataset (SEQs). A linear regression line is shown. The color gradient, ranging from blue to red, indicates the variation in the number of sequences. On the Y‐axis of all figures, the number of population clusters is displayed.

The most common groups of multicellular organisms Mollusca, Echinodermata, Crustacea (subphylum of Arthropoda phylum), Polychaeta (class of Annelida phylum), and Actinopterygii (class of Chordata phylum) were chosen for that analysis. Taxonomic‐based searches were performed in the popset database of the NCBI GenBank resource (Benson et al., 2018) among the sequence sets for population genetic studies. An important reason for the selection of these groups was their good coverage in GenBank, as well as the presence of homologous Leray *COI* marker fragments in NCBI. A total of 83 datasets were selected, with 9–20 sets for each group (Supplement B in Dryad data repository, Table S1) and at least 17 sequences in each dataset. Reduction of the retrieved sequences to the Leray fragment length was performed by aligning with reference datasets, which included 313 bp *COI* fragments with a retrieved reading frame and several complete *COI* gene sequences from each group. The sequence set was then joined with the reference sequence set in the MEGA program, translated given the mitochondrial code corresponding to the taxon, and aligned using ClustalW (Larkin et al., 2007) based on the Protein Weight Matrix BLOSUM with default parameters. If the alignment was successful (the Leray region was located within a fragment of the examined *COI* sequences between 130 and 236 amino acid sites of the complete translated gene sequence), the matrix was truncated according to the Leray fragment length. Subsequent analyses use the original dataset (also referred to as Original or Folmer for convenience), which has not undergone any processing, and the reduced or trimmed (Leray) dataset. The *haplotype* function of the pegas package (Paradis, 2010) was used to estimate the haplotypic variability of all the datasets obtained in this manner (both the original and trimmed). The number of population clusters in each dataset was estimated using the Geneland program (Guillot, 2008; Guillot et al., 2005) without prior information on the sampling location or other subdivision of the samples. The calculation was based only on the sites with SNPs, which were extracted from the sequence matrices using the SNP‐sites program (Page et al., 2016). Next, we used the console version of the PGDSpider program (Lischer & Excoffier, 2012) to convert the datasets into the format recommended by the Geneland authors (Guillot & Santos, 2010). During the preliminary stage, two independent MCMC runs were performed with the 100,000 and 500,000 generations, respectively, with 200 and 250 respective burn‐ins (discarded after the search). Sampling from the parameter space was performed every 100 generations. The maximum number of populations simulated during the search was set to 20 with the correlated frequency model. For datasets that showed differences in the number of determined populations between the first two runs, or runs that did not form a clear peak in the distribution of number of populations along the chain after burn‐in and also had a density below 0.5, an additional run was conducted using 1,500,000 generations to exclude possible under‐sampling during the search. The custom scripts providing simultaneous formatting and analysis of all datasets are given in the Supplement C of the Dryad data repository. Statistical processing and data visualization were performed in R (R Core Team, 2021). To compare the average number of clusters formed by the original (Original) and reduced (Leray) datasets (Figure 4a), we utilized the ggboxplot function of the ggpubr package (Kassambara, 2023) and assessed the reliability of the decrease in the number of clusters when reducing the size of the fragment using the Wilcoxon signed rank test with the wilcox. test function. Given the large variability in the number of sequences across different population datasets, we decided to evaluate the relationship between the number of sequences and the number of clusters formed by populations (Figure 4b). For this purpose, only original fragments were considered. In a similar manner, we examined the relationship between sequence length and the number of clusters identified based on them across different groups of organisms (Figure 4c). All figures were annotated and finalized in Inkscape (https://inkscape.org/).

## 3 RESULTS

### 3.1 Genotyping of hydrobionts from the mock communities

Assessing the hydrobionts for haplotypic variation in the Leray fragment (313 bp) showed that seven sequences of *H. octogrammus* contained three haplotypes in three variable sites. One of the haplotypes was present in five fish collected in Vityaz Bay, while the other two were found in fish collected in Vostok Bay. The sequence matrix of *P. dybowskii* contained one variable site with two haplotypes, without a clear association with sampling localities. In *P. latirostris* shrimp, we found two haplotypes located at one variable site. One of the haplotypes was found in a shrimp from Vityaz Bay, while the second haplotype was common to eight shrimps collected in Vostok Bay. Genotyping the Folmer fragment (~650 bp) revealed similar results, with the exception of *H. octogrammus*, in which five haplotypes were found in six variable sites (Table 1). Each individual was found to possess only one haplotype. The sequences obtained by this method revealed no signs of heteroplasmy or frameshift mutations.

### 3.2 Estimation of genetic diversity based on DNA metabarcoding data from the aquatic environments

After the filtering and demultiplexing procedure, the *COI* fragment had 1,197,893 reads, of which 513,152 were unique (Table 2). The negative PCR control detected 1308 reads (0.1% of the total). The search for taxonomic assignment based on the local reference, consisting of *COI* haplotypes of genotyped individuals from the mock communities, revealed 19 ASVs with 160,796 reads (117,866.22 ± 15,256.74^i^) (Tables 2 and 3). Of these, 8 ASVs corresponded to *H. octogrammus*, 9 ASVs belonged to *P. latirostris*, and 2 to *P. dybowskii*. Most of the reads (68%) accounted for ASVs assigned to *H. octogrammus* (110,628, or 69%). Regarding the original reference haplotypes, most of the reads (67.6%) also came from the three haplotypes of *H. octogrammus*. They formed a common cluster together with *H. octogrammus* AB755191 (Figure 4), with intraspecific variability equal to or less than 0.01. Five additional haplotypes of this species formed a separate phylogroup with divergence from 0.1 to 0.16. Four ASVs had a deletion of one nucleotide. In addition, DENOVO622 exhibited extra 2‐nuclotide deletion, leading to a shift in the reading frame (Figure 3). Moreover, all sequences displayed a non‐synonymous amino acid substitution (A–T). The *P. dybowskii* haplotypes accounted for 27% of all reads, which revealed similarities only to original reference haplotypes. *P. latirostris* had the least number of reads (6845, or 4.3%), but its nine ASVs were the most diverse, forming three phylogroups (Figure 2) and containing up to seven amino acid substitutions relative to the reference sequences (Figure 3). Furthermore, ASVs DENOVO3138 and DENOVO3088 exhibited stop codons within the reading frame. The divergence of additional ASVs from the cluster with the original haplotypes was between 0.026 and 0.121. The samples from the mock communities had 10,753 (Vityaz Bay) and 1288 (Vostok Bay) and reads. All additional ASVs of *P. latirostris* originated from Vityaz Bay, where most of the shrimp in the tank had been eliminated by the time of genotyping (see Materials and Methods section). Hap‐2 of this species, which was not detected in individuals from this location during the genotyping stage, is present in the same tank with a very high number of reads. Additional ASVs from *H. octogrammus* are almost not observed in artificial communities and are primarily found in individual eDNA samples. Thus, HO20‐6 Hap‐2 of this species carries additional ASVs DENOVO506 and DENOVO152, while hap1 carries DENOVO152 and DENOVO373. The data show that the original haplotypes are supported by at least 50 times more reads than their homologous additional ASVs. Some are at the detection threshold level (2–22 reads). It is interesting to note that the original haplotypes of different species are also supported by reads unevenly, and each haplotype of *P. latirostris* receives significantly fewer reads (154–714) compared to each original haplotype of the other two species (22,928–36,392).

**TABLE 2:** Number of reads corresponded to each of the aquatic DNA samples.

Sample or haplotype name	Total number of reads/number of reads assigned to reference haplotypes
PD20‐1	72,592/22,928
PD20‐3	103,077/19,395
PL20‐1	100,536/177
PL20‐2	174,597/719
HO20‐4	120,273/34,996
HO20‐6	117,520/34,129
HO20‐7	91,637/36,411
RA^a^	198,507/1288
LA^b^	217,846/10,753
Negative PCR control	1308/0
Total	1,197,893/160,796

^a^ Mock community based on samples from Vostok Bay.

^b^ Mock community based on samples from Vostok Bay.

**TABLE 3:** ASVs found based on the local reference database, indicating the number of reads.

ASV	Species	Vostok					Vityaz		LA^a^	RA^b^ Vost	Total
		PD_hap1	PD_hap2	HO_hap2	HO_hap3	PL_hap2	HO_hap1	PL_hap1			
PD20‐1 Hap‐1	*P. dybowskii*	22,928	6	0	0	5	0	0	156	44	23,139
PD20‐3 Hap‐2		0	19,389	0	0	0	0	0	459	336	20,184
HO20‐4 Hap‐1	*H. octogrammus*	0	0	24	7	0	34,201	23	4252	0	38,507
HO20‐6 Hap‐2		0	0	33,023	0	0	19	0	0	313	33,355
HO20‐7 Hap‐3		0	0	0	36,392	0	0	0	0	489	36,881
506		0	0	592	0	0	0	0	0	0	592
152^c^, ^d^		0	0	490	0	0	497	0	9	0	996
622^c^, ^d^		0	0	0	5	0	0	0	0	0	5
373^c^, ^d^		0	0	0	0	0	279	0	6	0	285
625^c^, ^d^		0	0	0	7	0	0	0	0	0	7
PL20‐1 Hap‐1	*P. latirostris*	0	0	0	0	0	0	154	7	0	161
PL20‐2 Hap‐2		0	0	0	0	714	0	0	5773	106	6593
1386		0	0	0	0	0	0	0	21	0	21
3056		0	0	0	0	0	0	0	6	0	6
3088^d^		0	0	0	0	0	0	0	15	0	15
3101		0	0	0	0	0	0	0	9	0	9
3138^d^		0	0	0	0	0	0	0	10	0	10
3142		0	0	0	0	0	0	0	22	0	22
3143		0	0	0	0	0	0	0	8	0	8

^a^ Mock community based on samples from Vityaz Bay.

^b^ Mock community based on samples from Vostok Bay.

^c^ Deletions detected.

^d^ Stop codon revealed.

The presence of additional ASVs with deep divergence to original *H. octogrammus* and *P. latirostris* haplotypes requires proof of their homology to these taxa. The availability of a complete nucleotide sequence reference database for family *Hexagrammidae* allowed us to determine whether additional ASVs belong to any of the known greenling species. When comparing the identified ASVs with the specified database, it was found that they occupy monophyletic cluster with *H. octogrammus* (Figure S2). The remaining four ASVs form a separate cluster occupying an intermediate position between the genera *Hexagrammos* and *Pleurogrammus*. Although a complete *COI* gene reference for the *Pandalus* genus is unavailable, our findings indicate that the additional ASVs do not correspond to any known species. Unlike the data obtained for *Hexagrammos*, these ASVs form a cohesive cluster with the original haplotypes (Figure S3).

The results of ASV condensation using the lulu program yielded six OTUs (Table 4). The species *P. dybowskii* and *P. latirostris* retained one haplotype per species. At the same time, the species *H. octogrammus* with rather high intraspecific variability retained three OTUs which are the reference haplotypes, as well as one of the haplotypes of the divergent cluster, carrying one nucleotide deletion.

**TABLE 4:** Results of the LULU package ASVs condensation.

OTU	PD_hap1	PD_hap2	HO_hap2	HO_hap3	PL_hap2	HO_hap1	PL_hap1	LA	RA
*H. octogrammus DENOVO152*	0	0	490	0	0	497	0	9	0
PD20‐1 Hap1 ON714502	22,928	6	0	0	5	0	0	156	44
PL20‐2 Hap 2 ON714508	0	0	0	0	714	0	0	5773	106
HO20‐4 Hap 1 ON714504	0	0	24	7	0	34,201	23	4252	0
HO20‐7 Hap 3 ON714506	0	0	0	36,392	0	0	0	0	489
HO20‐6 Hap 2 ON714505	0	0	33,023	0	0	19	0	0	313

*Note*: “RA” and “LA” correspond to the mock communities based on samples from the Vostok and Vityaz Bays, respectively.

### 3.3 Genetic variation of *COI* fragments from GenBank population datasets

The number of sequences in each dataset ranged from 17 to 350 (84.3 ± 5.5). The original length of the fragments ranged from 366 to 1153 bp (652 ± 13.7). Up to 11 groups based on the original fragment (3.2 ± 0.2) and up to 9 groups based on the reduced fragment (2.4 ± 0.2) were identified by the analysis of intraspecific structure. Around 27.7% of the original datasets (23 sets) showed no intraspecific structure, while 14.5% (12 sets) of the Leray datasets did (Table S1). In total, 79 datasets gave a reliable determination of the number of clusters (density value of 80% or higher according to calculations on the non‐reduced set), while for the remaining four (*Johora singaporensis* (Ng, 1986) (Crustaceans), *Gobiesox adustus* (Jordan & Gilbert, 1882), *Rhinogobius formosanus* (Oshima, 1919), and *Scleropages formosus* (Müller & Schlegel, 1840) (Fishes)), even a repeated run using more generations did not increase the reliability of results (density value was from 24 to 60%). A significant decrease in the number of clusters was observed when using the reduced dataset, according to the results of the Wilcoxon pairwise test (W = 1002, *p* < .001, n = 83, one‐tailed, Figure 4a). In contrast, one species (*Callinectes sapidus* (Rathbun, 1896) (Crustaceans)) showed the opposite trend, although it was associated with a decrease in the confidence of the intraspecific analysis (Table S1). A decrease in validity was also recorded for the species *Laophontella armata* (Willey, 1935) (Crustaceans) when using the reduced dataset. Aside from these two cases, the reduction did not cause a decrease in validity for the other datasets (Table S1). We also discovered a moderate positive correlation between the number of sequences and the number of intraspecific clusters, which proved to be significant (*r* = .46, *p* < .0001, Figure 4b). No statistically significant correlation was found between the length of sequences in the dataset and the number of clusters for most groups of organisms, considering the type of fragment (Figure 4c). Only in the Mollusca group, under the same conditions of significance, was a moderate positive correlation (0.334) detected.

One of the species, *Paracentrotus lividus* (Lamarck, 1816) (Echinodermata), had a noticeably shorter Leray region among the reduced datasets based on the amino acid sequence alignment results (Figure S4). The Leray region in this species is 247 bp long, which is caused by a 66 bp deletion located between 160 and 183 positions and affecting 22 amino acid residues. No other data on fragment length polymorphism were found in the analyzed datasets.

## 4 DISCUSSION

This study explores the possibilities and limitations of rapidly assessing genetic variability among abundant marine species using a standardized *COI* metabarcode under experimental conditions. It also tests the hypothesis that NUMTs, represented by additional related ASVs, may compete for primer binding during the PCR. In addition, using the data retrieved from GenBank, we examined the extent to which the number of detectable populations (clusters) is affected by the use from of the *COI* barcode versus metabarcode.

Recent studies show that environmental DNA can provide detailed information on species‐specific haplotype diversity, although currently only few studies have successfully used this approach to obtain population‐level genetic data (Adams et al., 2022; Andres et al., 2021; Elbrecht et al., 2018; Sigsgaard et al., 2017; Tsuji, Maruyama, et al., 2020; Tsuji, Shibata, et al., 2020). Factors such as sequencing errors that lead to false positives, as well as limitations in the understanding of the processes that affect the rate of environmental DNA production can affect the validity of the results (Eble et al., 2020). It is possible that primer bias may also affect the results (Shu et al., 2021). Moreover, fundamental differences in primer design approaches for target species detection and metabarcoding surveys cause further challenges (Freeland, 2017). Typically, in such studies at least 30,000 reads per sample are required to reach a plateau at the number of OTUs (Dully et al., 2021). At the same time, there is evidence that even for individually designed markers for target species, the number of detectable haplotypes using eDNA can be lower than their actual number (Adams et al., 2022).

A study of haplotype diversity among *Plecoglossus altivelis altivelis* fishes using the mitochondrial *D‐loop* marker showed high accuracy in determining variation using ASV noise reduction algorithms, which eliminated false positives (Tsuji, Miya, et al., 2020). Further studies in this area showed that the haplotype diversity estimated from invasive screening was lower compared to estimates obtained using environmental DNA at all screening sites (Tsuji, Maruyama, et al., 2020; Tsuji, Shibata, et al., 2020). Non‐invasive DNA sampling was found to be prone to overestimate intraspecific genetic diversity, while invasive sampling led to underestimations. Similarly, the results varied depending on which noise reduction algorithm was used (Tsuji, Maruyama, et al., 2020).

Another study indicated that the analysis of environmental DNA yields a result close to the estimated intraspecific diversity of the entire population. In addition, it has been observed that the discrepancy between the number of haplotypes estimated based on DNA extracted from tissues compared to that estimated using environmental DNA is most likely due to differences in sample coverage (Tsuji, Shibata, et al., 2020). The above‐mentioned studies used the *D‐loop* as a marker, which is a non‐coding region with variability higher than most other mitochondrial and some nuclear markers.

It is known that markers encoding proteins, including *COI*, have a lower level of mutations, and may tend to increase the detection accuracy due to noise suppression. Contrary to the use of individual markers for target species, our study highlights a method for mass parallel biodiversity monitoring using a universal set of primers (Geller et al., 2013; Leray et al., 2013; Wangensteen et al., 2018). It also addresses the bias introduced by PCR‐based methods (Yang et al., 2021), but in this case, it specifically focuses on haplotypic diversity. While *COI*'s lower mutation rate compared to other markers like the *D*‐loop may limit its sensitivity in detecting fine‐scale intraspecific diversity, its extensive use in population genetics and phylogeographic studies lends a robust framework for comparative analyses across taxa (Turon et al., 2020). Incorporating additional markers and considering factors, such as primer choice and sample size, can enhance the detection of haplotype diversity and address the limitations associated with *COI* metabarcoding (Clarke et al., 2017; Drummond et al., 2015; Turon et al., 2020).

In addition to the original *COI* haplotypes identified through individual genotyping of aquatic organisms, we discovered additional ASVs. These ASVs are not merely homologous to the original ones but also exhibit a high degree of similarity (Figure 2, Figures [Link], [Link], Table 3). However, we could not a priori identify them as pseudogenes.

To clarify the nature of additional ASVs and their origins, the document outlines assumptions regarding (1) PCR and sequencing errors and tags jumps, suggesting artifacts may falsely inflate diversity; (2) contributions from duplicated mitochondrial regions, indicating some ASVs may derive from within‐species genomic variations; (3) cryptic or previously overlooked species diversity, implying some ASVs could represent distinct species not yet recognized; and (4) NUMTs or pseudogenes, suggesting some ASVs might originate from nuclear sequences mimicking mitochondrial DNA, rather than genuine mitochondrial diversity.

DNA metabarcoding studies are known to be susceptible to artifacts such as tag jumps and PCR errors, which can lead to incorrect sequence assignments and inflated diversity estimates (Acinas et al., 2005; Rodriguez‐Martinez et al., 2022; Schnell et al., 2015; Zizka et al., 2019). In addition to trivial incorrect assignment to samples, tag jumps can be caused by blunt‐ending during library build and chimera formation during PCR amplification (Rodriguez‐Martinez et al., 2022; Schnell et al., 2015). Using twin‐tagged primers which we applied in our study, can help prevent false sequence assignments due to tag jumps (Carøe & Bohmann, 2020). However, this method also increases cost and workload. The occurrence of identical PCR or sequencing errors with high coverage in different samples is unlikely (see ASV 152 in HO_hap1 and HO_hap2 samples). Moreover, to avoid PCR artifacts, we used both widely used commercial kits during the PCR stage and the PCR‐free library preparation technique.

Currently, there is no information on the complete sequences of the mitochondrial genome of *P. latirostris*, but a closely related species of this genus, *Pandalus borealis* Krøyer, 1838, does not exhibit such structural features (Viker et al., 2006). In fish, duplication of mitochondrial genome fragments is generally a rare phenomenon (however, see the flatfish (Li et al., 2015)), and species close to *H. octogrammus* also do not exhibit such features (Ji et al., 2020). The presence of cryptic diversity cannot be excluded for *P. latirostris*, not only because of the lack of a complete reference library for the genus (Figure S3), but also because several specimens were consumed by others in the artificial communities during the experiment (see Materials and Methods). Accordingly, at least a part of the ASVs of this species can be attributed to unidentified, yet present haplotypes in the artificial communities (Figure 2, Table 3). Comparing our haplotypes with sequences from the sites of the original species description (Figure 2, id. DRR) reveals that the intraspecific variation of *P. latirostris*, based on the utilized marker, is consistent throughout its range (from Notsuke Bay to Peter the Great Gulf), with variation not exceeding 0.003 (Table 5). This may also suggest the possibility that these individuals may have derived from a common haplogroup. It is important to note that this species lacks planktonic larvae and exhibits distinct population structure even across small ranges, as demonstrated by more variable markers (Azuma & Chiba, 2018). Hence, we are committed to the view that ASVs diverging from the reference ones by at least 0.026 *p*‐distance value (Figure 2, Table 5) may be considered as having originated from a different type of phenomenon. These are highly likely to be nuclear copies of mitochondrial sequences or NUMTs, which is a type of pseudogenes (Hazkani‐Covo et al., 2010; Marshall & Parson, 2021). The divergent ASVs (Figure 2, Tables 5 and 6) are characterized either by non‐synonymous nucleotide substitutions and stop codons within reading frame (*P. latirostris* and *H. octogrammus*, Figure 3a,b) or a shift in the reading frame (*H. octogrammus*, Figure 3b), hence can be attributed to pseudogenes, having previously excluded other interpretations of their origin.

**TABLE 5:** The values of genetic *p*‐distances calculated between ASVs related to *COI* Leray region of *P. latirostris*.

No	Sequence ID	1	2	3	4	5	6	7	8	9	10	11	12
1	PL20‐1 Hap‐1 ON714507												
2	PL20‐2 Hap‐2 ON714508	0.003											
3	DRR001118	0.003	0										
4	DRR001119	0.003	0	0									
5	DRR001120	0.003	0	0	0								
6	DRR001121	0.003	0	0	0	0							
7	DENOVO13863	0.026	0.022	0.022	0.022	0.022	0.022						
8	DENOVO3138	0.038	0.042	0.042	0.042	0.042	0.042	0.045					
9	DENOVO3143	0.054	0.058	0.058	0.058	0.058	0.058	0.061	0.022				
10	DENOVO3088	0.054	0.058	0.058	0.058	0.058	0.058	0.061	0.035	0.035			
11	DENOVO3101	0.061	0.064	0.064	0.064	0.064	0.064	0.067	0.042	0.042	0.006		
12	DENOVO3056	0.067	0.07	0.07	0.07	0.07	0.07	0.073	0.048	0.048	0.013	0.006	
13	DENOVO3142	0.121	0.125	0.125	0.125	0.125	0.125	0.121	0.118	0.128	0.128	0.134	0.141

**TABLE 6:** The values of genetic *p*‐distances calculated between ASVs related to *COI* Leray region of *H. octogrammus*.

No	Sequence ID	1	2	3	4	5	6	7	8
1	*H. octogrammus* AB755191								
2	HO20‐7 Hap‐3 ON714506	0							
3	HO20‐4 Hap‐1 ON714504	0.006	0.006						
4	HO20‐6 Hap‐2 ON714505	0.01	0.01	0.003					
5	DENOVO506	0.1	0.1	0.097	0.1				
6	DENOVO152	0.106	0.106	0.103	0.106	0.013			
7	DENOVO622	0.106	0.106	0.103	0.106	0.019	0.006		
8	DENOVO373	0.113	0.113	0.11	0.113	0.019	0.006	0.006	
9	DENOVO625	0.116	0.116	0.113	0.116	0.023	0.01	0.01	0.01

In general, it is rather difficult to differentiate NUMTs from sequences of the mitochondrial DNA (Hazkani‐Covo et al., 2010; Nugent et al., 2020; Porter & Hajibabaei, 2021; Triant & DeWoody, 2007). In this respect, it is much easier to work with model organisms, as reference genomes are available (Marshall & Parson, 2021), as well as try to prevent accumulation of NUMTs at the experimental stage (Wang et al., 2019). NUMTs are not typically detected during Sanger sequencing of organismal DNA samples because of their small number in the amplicon pool (Hebert et al., 2004; Schultz & Hebert, 2022), unlike the situation with eDNA. In our case (with the exception of ASVs with deletions carrying a reading frame shift, Figure 3b), we can be satisfied with the exclusion method only if a pre‐collected complete barcode DNA reference library for the species in question is available. At this point, we can expect that almost all phylogeographic studies based on environmental DNA metabarcoding are subject to “survivorship bias,” where only what has passed selection by primers and sequencing coverage is analyzed. It cannot be excluded that our study also suffers from this drawback. Processing the obtained ASVs using the Lulu feature essentially produced the expected results. For example, we showed that sufficiently divergent NUMTs are not eliminated, but remain as a separate ASVs (Figure 2, Tables 4, 5, 6), especially when they supported by sufficient number of reads. However, there is no proper database of pseudogenes, which also relates to the issue of “survivorship bias.” In this respect, we agree with the perspective that it is necessary to maintain a pseudogene database in addition to the well‐curated barcode reference library (Schultz & Hebert, 2022).

A recent paper examining the expected frequency of NUMTs in various marine animals indicated that it is precisely the use of *COI* Leray that may pose the highest risk of detecting NUMTs in metabarcoding studies (Schultz & Hebert, 2022). This is because more than 58% of the pseudogenes identified in the study were of lengths up to 300 bp, although such research is more similar methodologically to that based on a PCR‐free approach or metagenomics (Singer et al., 2020) rather than metabarcoding. It should be emphasized that the results obtained in our work are most likely valid only for metabarcoding, where PCR causes a bias resulting in misrepresentation of the haplotypic diversity of the environmental samples (Table 3). Based on the analyzed data, the assumption about the possible competitive advantage of NUMTs over the original mitochondrial DNA haplotypes of three aquatic organisms during PCR is not supported. However, it is important to note that this does not negate the issue of pseudogene acquisition through PCR based on primers to short genome fragments (but see Balciuniene & Balciunas, 2019), and their influence should be expected, with appropriate tools used for their detection (Haran et al., 2015; Marshall & Parson, 2021; Ring et al., 2018).

The calculations based on the data retrieved from GenBank do not allow us to formulate any recommendations for correcting works to detect genetic diversity using environmental DNA metabarcoding (Figure 4). These data also need to be considered as potentially biased due to the «survivorship bias», as there is a higher likelihood that marker data, which can actually reflect the population structure of a given species, are deposited in the GenBank. However, a natural variation in fragment length can quite rarely be expected, which can be used to correct for filtering fragments by length during computation without decreasing the reliability of the results. The number of population‐genetic clusters calculated in this work is a rather conservative measure, and is not customized for a particular dataset with the choice of the exact model. However, it is evident that using the metabarcode instead of barcode causes a reduction in the number of identifiable populations, as in our case one cluster on average was dropped. For the sets that did not exhibit a decrease, one cannot detect any pattern other than the intuitive conclusion that they were not affected by length reduction because of the strong divergence, and the random concentration of all information within the metabarcode region.

## AUTHOR CONTRIBUTIONS

**S. V. Turanov:** Conceptualization (lead); data curation (lead); formal analysis (lead); funding acquisition (equal); investigation (lead); methodology (lead); project administration (lead); resources (equal); software (lead); supervision (lead); validation (lead); visualization (lead); writing – original draft (lead); writing – review and editing (lead). **O. A. Rutenko:** Funding acquisition (equal); investigation (supporting); methodology (supporting); project administration (supporting); resources (supporting); validation (supporting); writing – review and editing (supporting). **M. A. Koltsova:** Data curation (supporting); formal analysis (supporting); investigation (supporting); methodology (supporting); resources (supporting); software (supporting); visualization (supporting); writing – original draft (supporting).

## FUNDING INFORMATION

The sequencing duties in this study were partially supported by the Ministry of Science and Higher Education of the Russian Federation in the framework of the Federal Project #13.1902.21.0012 (agreement no. 075‐15‐2020‐796).

## CONFLICT OF INTEREST STATEMENT

The authors declare no conflicts of interest.

## Supporting information


Figure S1

Figure S2

Figure S3

Figure S4

Table S1

## Data Availability

The reads are openly available in the NCBI Sequence Read Archive under SRR29409451 run number (BioProject PRJNA899333). Scripts and supporting information are publicly available at the Dryad data repository https://doi.org/10.5061/dryad.sf7m0cgfk.

## Endnote

^i^
Hereinafter, the values are the mean ± standard deviation.
