# Supplementary material for: Experimental evaluation of genetic variability based on DNA metabarcoding from the aquatic environment: Insights from the Leray COI fragment
Source: Ecol Evol. 2024 Jul 4;14(7):e11631. doi: 10.1002/ece3.11631 (PMC11222756; doi:10.1002/ece3.11631)
Supplement: Supplementary file 1 — Figure S1 [file ECE3-14-e11631-s004.pdf]

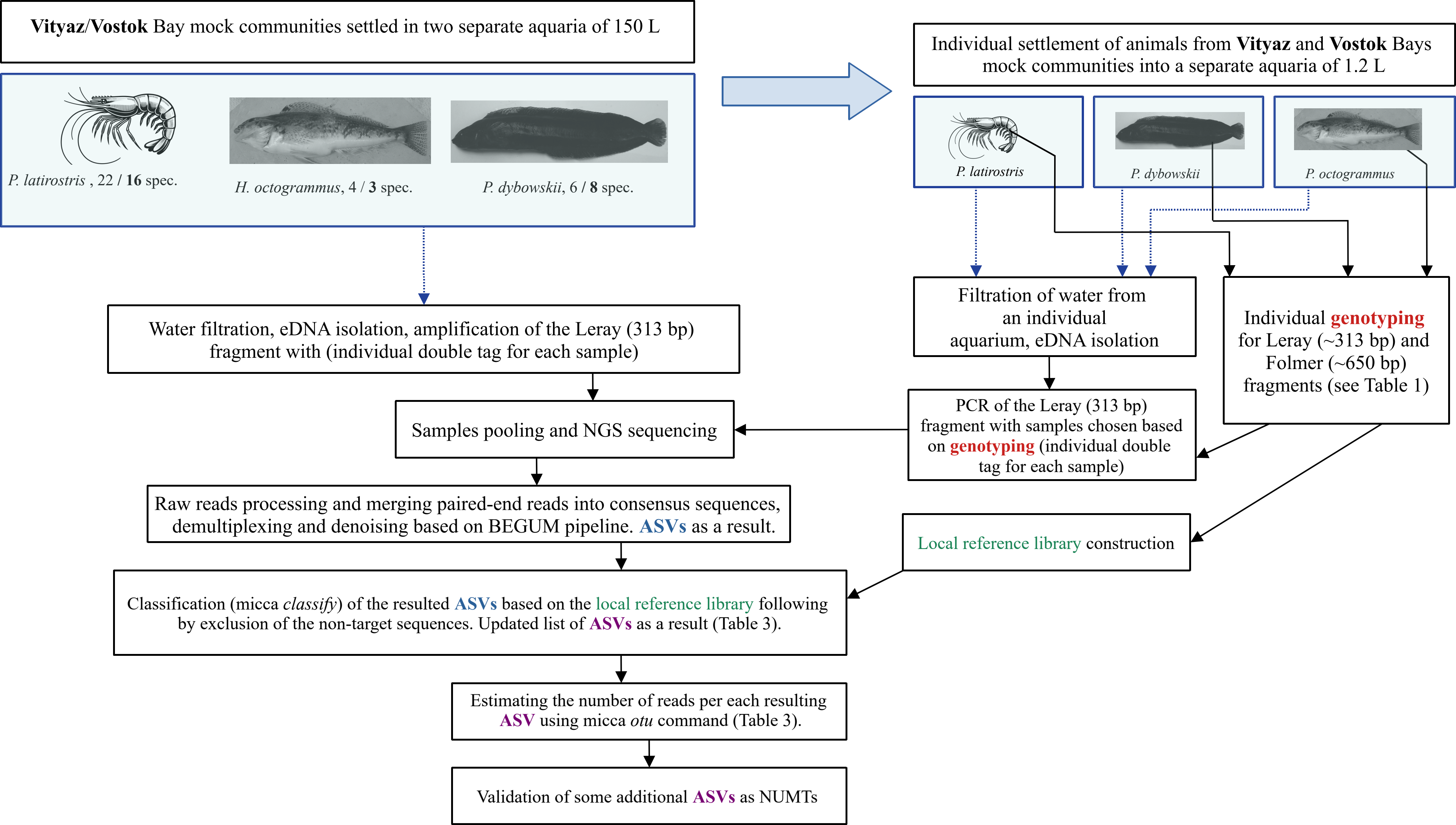
Vityaz/Vostok Bay mock communities settled in two separate aquaria of 150 L
P. latirostris , 22 / 16 spec.
H. octogrammus, 4 / 3 spec.
P. dybowskii, 6 / 8 spec.
Individual settlement of animals from Vityaz and Vostok Bays mock communities into a separate aquaria of 1.2 L
P. latirostris
P. dybowskii
P. octogrammus
Water filtration, eDNA isolation, amplification of the Leray (313 bp) fragment with (individual double tag for each sample)
Filtration of water from an individual aquarium, eDNA isolation
Individual genotyping for Leray (~313 bp) and Folmer (~650 bp) fragments (see Table 1)
Samples pooling and NGS sequencing
PCR of the Leray (313 bp) fragment with samples chosen based on genotyping (individual double tag for each sample)
Raw reads processing and merging paired-end reads into consensus sequences, demultiplexing and denoising based on BEGUM pipeline. ASVs as a result.
Local reference library construction
Classification (micca classify) of the resulted ASVs based on the local reference library following by exclusion of the non-target sequences. Updated list of ASVs as a result (Table 3).
Estimating the number of reads per each resulting ASV using micca otu command (Table 3).
Validation of some additional ASVs as NUMTs
