# Supplementary figures and images for: Experimental evaluation of genetic variability based on DNA metabarcoding from the aquatic environment: Insights from the Leray COI fragment
Source: Ecol Evol. 2024 Jul 4;14(7):e11631. doi: 10.1002/ece3.11631 (PMC11222756; doi:10.1002/ece3.11631)

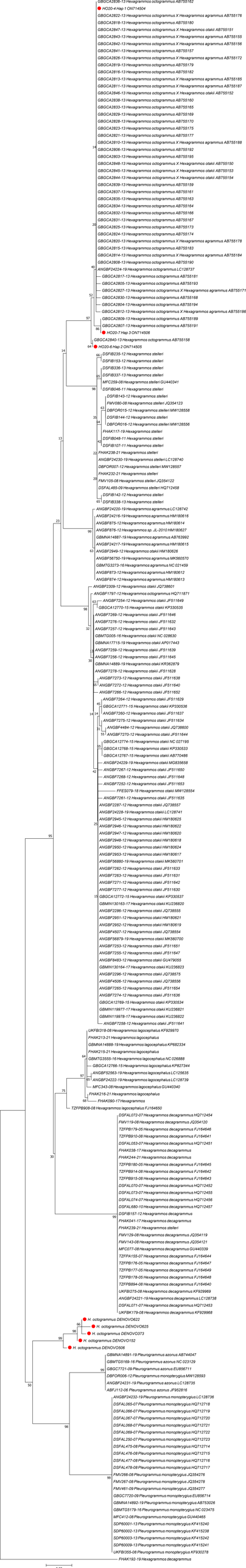

Supplement: Supplementary file 2 — Figure S2 [file ECE3-14-e11631-s005.pdf]

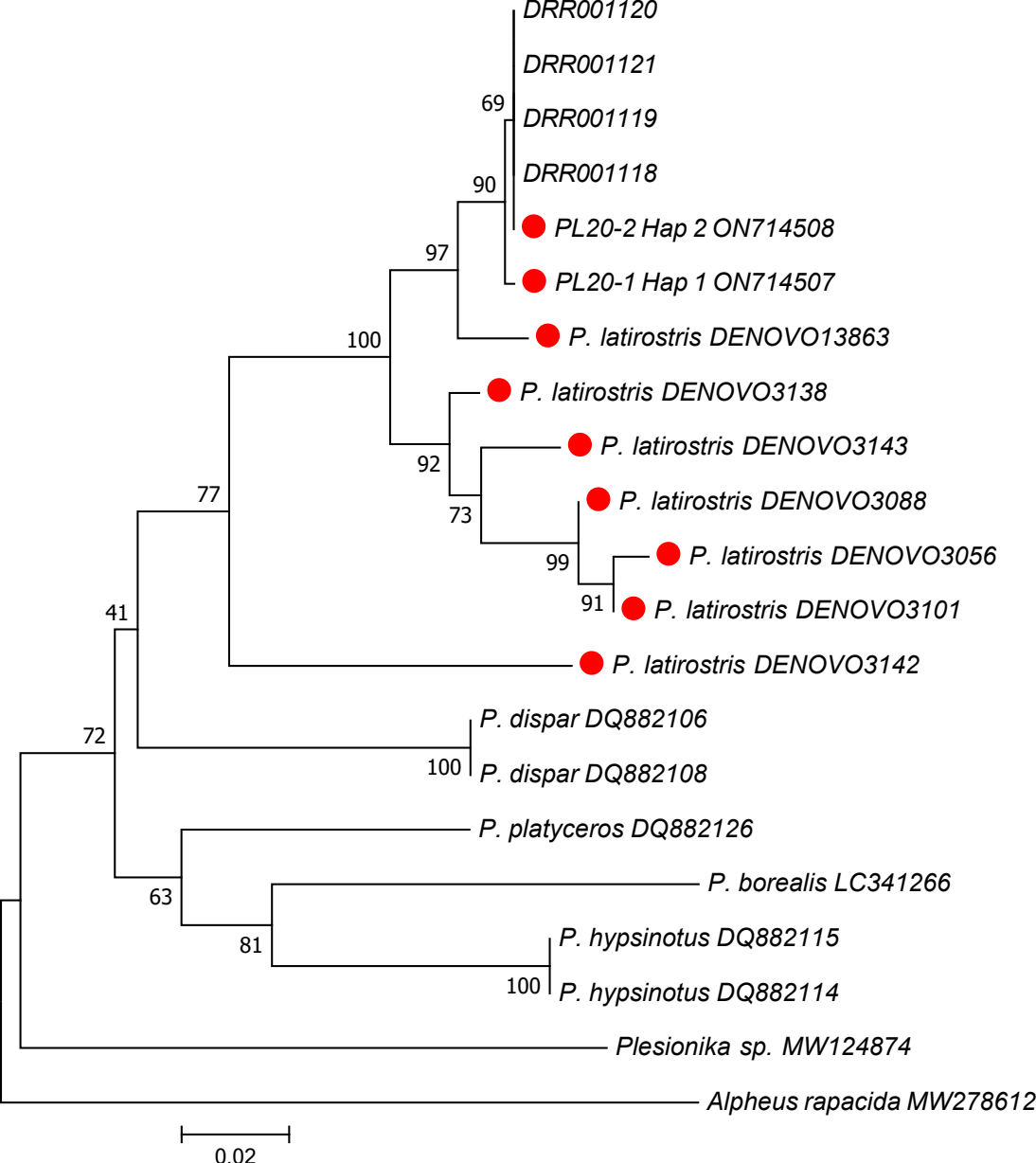

Supplement: Supplementary file 3 — Figure S3 [file ECE3-14-e11631-s001.pdf]
