# Supplementary material for: Experimental evaluation of genetic variability based on DNA metabarcoding from the aquatic environment: Insights from the Leray COI fragment
Source: Ecol Evol. 2024 Jul 4;14(7):e11631. doi: 10.1002/ece3.11631 (PMC11222756; doi:10.1002/ece3.11631)
Supplement: Supplementary file 4 — Figure S4 [file ECE3-14-e11631-s003.pdf]

```
                                  128                             161                  182
MN198190 Ph. liuwutiensis         IYP|PLSSNIAHAGGSVDLAIFSLHLAGASSILASINFITTIINMRSSGISFDRLPLFIWSVFITA
KJ680292 H. mammillatus           IYP|PLSSNIAHAGGSVDLAIFSLHLAGASSILASINFITTIINMRTPGMSFDRLPLFVWSVFVTA
NC_053361 C. papposus             MYP|PLSSGLAHAGGSVDLAIFSLHLAGASSILASINFITTVINMRTPGITFDRLPLFVWSVFVTA
KC706832 Asterina sp. DL_230A     ---|?LSSGLAHAGGSVDLAIFSLHLAGASSILASINFITTVINMRTPGISFDRLPLFVWSVLVTA
KC706833 S. horrens BMOO-02294    ---|?LSSNIAHAGGSVDLAIFSLHLAGASSILASINFITTIINMRTPGVTFDRLPLFVWSVFITA
KU496263 A. yairi JOD_0202        ---|?LSSSLAHAGGSVDLAIFSLHLAGASSILASINFITTVINMRTPGISFDRLPLFVWSVFVTA
MG063890 P. lividus P1-F01        ---|?LSSNIAHAGGSVDLAIFSLHLAGASSILP----------------------LFVWSVFVTA
MG063890 P. lividus P1-F05        ---|?LSSNIAHAGGSVDLAIFSLHLAGASSILP----------------------LFVWSVFVTA
MG063890 P. lividus P1-F06        ---|?LSSNIAHAGGSVDLAIFSLHLAGASSILP----------------------LFVWSVFVTA

                                  193                                       239
MN198190 Ph. liuwutiensis         FLLLLSLPVLAGAITMLLTDRNINTTFFDPAGGGDPILFQHLF|WFFG
KJ680292 H. mammillatus           FLLLLSLPVLAGAITMLLTDRNINTTFFDPAGGGDPILFQHLF|WFFG
NC_053361 C. papposus             FLLLLSLPVLAGAITMLLTDRNINTTFFDPAGGGDPILFQHLF|WFFG
KC706832 Asterina sp. DL_230A     FLLLLSLPVLAGAITMLLTDRNVNTTFFDPAGGGDPILFQHLF|----
KC706833 S. horrens BMOO-02294    FLLLLSLPVLAGAITMLLTDRNINTTFFDPAGGGDPILFQHLF|----
KU496263 A. yairi JOD_0202        FLLLLSLPVLAGAITMLLTDRNVNTTFFDPAGGGDPILFQHLF|----
MG063890 P. lividus P1-F01        FLLLLSLPVLAGAITMLLTDRNINTTFFDPAGGGDPILFQHLF|----
MG063890 P. lividus P1-F05        FLLLLSLPVLAGAITMLLTDRNINTTFFDPAGGGDPILFQHLF|----
MG063890 P. lividus P1-F06        FLLLLSLPVLAGAITMLLTDRNINTTFFDPAGGGDPILFQHLF|----
```
